# Increased reaction times and reduced response preparation already starts at middle age

**DOI:** 10.3389/fnagi.2014.00079

**Published:** 2014-04-28

**Authors:** Ria Wolkorte, Janine Kamphuis, Inge Zijdewind

**Affiliations:** Department of Neuroscience, University Medical Center Groningen, University of GroningenGroningen, Netherlands

**Keywords:** choice reaction time, accuracy, aging, muscle fatigue, sex, motor preparation

## Abstract

Generalized slowing characterizes aging and there is some evidence to suggest that this slowing already starts at midlife. This study aims to assess reaction time changes while performing a concurrent low-force and high-force motor task in young and middle-aged subjects. The high-force motor task is designed to induce muscle fatigue and thereby progressively increase the attentional demands. Twenty-five young (20–30 years, 12 males) and 16 middle-aged (35–55 years, 9 males) adults performed an auditory two-choice reaction time task (CRT) with and without a concurrent low- or high-force motor task. The CRT required subjects to respond to two different stimuli that occurred with a probability of 70 or 30%. The motor task consisted of index finger abduction, at either 10% (10%-dual-task) or 30% (30%-dual-task) of maximal voluntary force. Cognitive task performance was measured as percentage of correct responses and reaction times. Middle-aged subjects responded slower on the *frequent* but more accurately on the *infrequent* stimuli of CRT than young subjects. Both young and middle-aged subjects showed increased errors and reaction times while performing under dual-task conditions and both outcome measures increased further under fatiguing conditions. Only under 30%-dual-task demands, an age-effect on dual-task performance was present. Both single- and dual-task conditions showed that already at mid-life response preparation is seriously declined and that subjects implement different strategies to perform a CRT task.

## INTRODUCTION

With increasing age cognitive performance slows down (Verhaeghen and Salthouse, 1997; Der and Deary, 2006), including cognitive processes essential for motor performance (Yordanova et al., 2004). Additionally, performance of motor tasks becomes less automatic and requires increased attentional demands in older subjects (57–75 years; Heuninckx et al., 2005; Wu and Hallett, 2005; Voelcker-Rehage and Alberts, 2007). A classic method to study the distribution of attentional and processing capacity is the dual-task paradigm (Pashler, 1994). During a dual-task attention needs to be allocated to two different tasks that are performed simultaneously. If the two tasks are simple and do not require shared input, information processing or output modalities, the two tasks can be performed concurrently without a decline in performance in either of the two tasks. However, performance of two concurrent tasks often results in a performance decline; if the two tasks do not share input- or output modalities then quantification of this interference can be used to address the distribution of attentional resources between the two tasks (Welford, 1988; Pashler, 1994). Given the observation that attentional demands for performing a single motor task already increases with age (Mattay et al., 2002; Ward and Frackowiak, 2003; Heuninckx et al., 2005; Wu and Hallett, 2005) we expected that for older subjects performing a cognitive-motor dual-task would be even more difficult. Several studies indeed found a decline in cognitive-motor task performance with age (60–90 years; Crossley and Hiscock, 1992; Voelcker-Rehage and Alberts, 2007; Fraser et al., 2010) and a meta-analysis by Verhaeghen et al. (2003) revealed that dual-task costs with aging were greater than the general age-related slowing in reaction times.

Previous studies in our group on young subjects showed a decline in performance on a cognitive task during a concurrently performed motor task, and additionally that the decline became stronger when the motor task was fatiguing (Lorist et al., 2002; Zijdewind et al., 2006). In those as well as in the present study muscle fatigue is defined as an increased effort to maintain a desired force level (demonstrated by an increase in electromyographic activity, EMG) and a decline in maximal force generating capacity followed by the subsequent inability to maintain a submaximal target force. During a sustained submaximal contraction, muscle fibers become fatigued and an increase in voluntary drive is necessary to maintain the submaximal force (van Duinen et al., 2007). We expected that older subjects would have more difficulties in performing a cognitive-motor dual-task and that fatigue would contribute to a further deterioration of the dual-task performance. In other words, fatigue was used as an extra stressor to highlight possible age-related differences in dual task performance.

In our cognitive-motor dual-task (Lorist et al., 2002; Zijdewind et al., 2006), we used a choice reaction time task (CRT) as the secondary, cognitive task. In the CRT we used two stimuli (a high-pitched and a low-pitched tone). One of the stimuli of the CRT occurred more often than the other stimulus (Goodin et al., 1990; Miller, 1998) and subjects were thus primed to prepare a response to the frequent stimulus. Preparing for the frequent stimuli leads to faster reaction times for this stimulus, albeit with more erroneous responses for the infrequent stimulus. It is therefore expected that subjects who prepared more for the frequent stimuli would demonstrate larger differences between reaction times and accuracy on frequent and infrequent stimuli (Goodin et al., 1990; Miller, 1998). Furthermore, we expected that responding to the infrequent stimulus would become more difficult and that, therefore, this stimulus would be more sensitive to changes in attention.

Previous reaction time studies showed that older subjects choose to maintain accuracy over speed in a dual-task condition (Rabbitt, 1979; Welford, 1988; Smith and Brewer, 1995). It is, therefore, conceivable that older subjects would be less willing to prepare for frequent stimuli (Cuypers et al., 2013) and thus show smaller differences in reaction times and accuracy than young subjects.

Most experiments have been performed on young (<35 years) and older subjects (>65 years). Neuroimaging studies, however, indicate that age-related changes can already appear in late midlife (Liu et al., 2003; Hartley et al., 2011; Garrett et al., 2012). Whether differences in dual-task costs and preparation are already manifest in middle-aged adults is still uncertain, although the results of Crossley and Hiscock (1992) suggest that effects may already appear in this age group (41–65 years). As middle-aged subjects are more likely to be actively involved on the work floor and considering the widespread use of electronic devices that require high cognitive and motor demands it is important to also investigate age-related dual-task changes in this age-group.

Therefore, it was the aim of the present study to investigate differences in response preparation and dual-task costs in young and middle-aged adults. Furthermore, we induced muscle fatigue during the dual-task to further increase the attentional load and to evoke additional dual-task interference in both young and middle-aged adults. We expected less response preparation and more dual task costs in middle-aged subjects. Furthermore, we expected the motor task to be performed adequately in both age groups, with only little dual-task costs on the motor task (Lorist et al., 2002). However, we expect an additional attentional cost in the middle-aged subjects what would result in an additional decline in cognitive performance in the middle-aged group without a significant difference in the performance on the fatiguing motor task. During the fatiguing dual task, we expected a further increase in dual task costs in both age-groups.

## MATERIALS AND METHODS

### PARTICIPANTS

We used data obtained in 25 young (mean 23 years, range 20–29, 12 males) and 16 middle-aged (mean 46 years, range 35–55, 9 males) adults. All subjects were right-handed according to the Edinburgh Handedness Questionnaire (range 38–100; Oldfield, 1971), had normal or corrected-to-normal vision and none reported hearing deficits. Level of education was determined based on the “standaard onderwijsindeling 2006,” (SOI 2006, CBS; the Dutch version of the ISCED). No difference was found in level of education between the age groups (*p* = 0.20). All participants gave their informed consent before participation. The study was approved by the University Medical Center Groningen medical ethical committee and was in conformance with the standard set out in the WMA Declaration of Helsinki (2008).

### EXPERIMENTAL SETUP

The experimental setup and tasks largely followed the methods as described by Lorist et al. (2002).

#### Force and electromyographic recordings during the motor task

The motor task consisted of abduction of the right index finger. Participants sat at a table with their lower arms resting on the table. The forearm of the right hand was stabilized with a splint halfway between pronation and supination. Digits three, four and five were constrained with a plastic plate and the thumb was fixated with a Velcro strap. The proximal interphalangeal joint of the index finger was taped against a wedge, connected to a force transducer. For details on the set-up, see also Zijdewind and Kernell (1994). The force signal was amplified and recorded at a sampling rate of 500 Hz. EMG was recorded with sintered Ag/AgCl electrodes located over the right first dorsal interosseus muscle (FDI). EMG was amplified 500 times, filtered between 8 and 1000 Hz, and recorded at a sampling frequency of 2000 Hz. Data was recorded and analyzed with a PC equipped with a data-acquisition interface and the accompanying software Spike2 v7 (1401 Power, Cambridge Electronic Design, Cambridge, UK). During the experiment, participants were given force feedback on a computer screen located approximately one meter in front of the subject. A target line showed the force that participants were required to deliver. A second line, in a different color, showed the actual produced force in real-time.

#### Set-up of the cognitive task

A response box was placed in front of the participant at a comfortable position so that the participant could have his right index finger placed in the force set-up and simultaneously respond with his left hand, positioned on the response box. The cognitive task consisted of an auditory CRT. Loudspeakers placed in front of the participant on both the left and right side produced either a low (500 Hz) or a high (900 Hz) pitched tone at a level of 70 dBA. The tones were of 50 ms duration with an inter-stimulus interval of 1100–1300 ms. The participants were instructed to respond as quickly and as accurately as possible by pressing a button on a serial response box with their left index or middle finger. E-Prime software was used to present the stimuli and record the responses (Psychology Software Tools, Inc., Sharpsburg, PA, USA). The expected response (index- or middle finger) to high and low tones was randomized between participants. The probability of one of the tones was more frequent (70%) than the other tone (30%). Which tone was presented more frequent was randomized between participants. Participants were informed that the first stimulus of every block was always the frequent stimulus.

### EXPERIMENTAL TASKS

**FIGURE 1:**
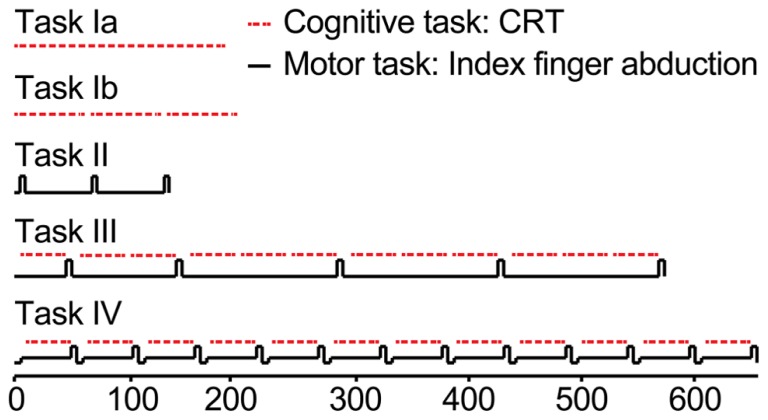
**Schematic overview of the protocol.** Red lines represent the cognitive task, black lines the motor task. Task Ia, practice of the choice reaction time task (CRT), 150 consecutive stimuli. Task Ib, practice of the choice reaction time task (CRT), 3 times 50 stimuli. Task II, right index finger abduction, determination of maximal voluntary force (MVC). Task III, Single CRT-task. Responding to the auditory stimuli as fast and as accurate as possible. Task IV, Dual task. One session the dual-task at 30% of cMVC (30%-dual-task), one session the dual-task at 10% of cMVC (10%-dual-task). All subjects performed three sessions. In session 1, all tasks were practiced; session 2 and 3 were the experimental sessions in which Task I to III were similar for the two sessions and Task IV was alternated between the 10 or the 30%-dual-task.

**FIGURE 2:**
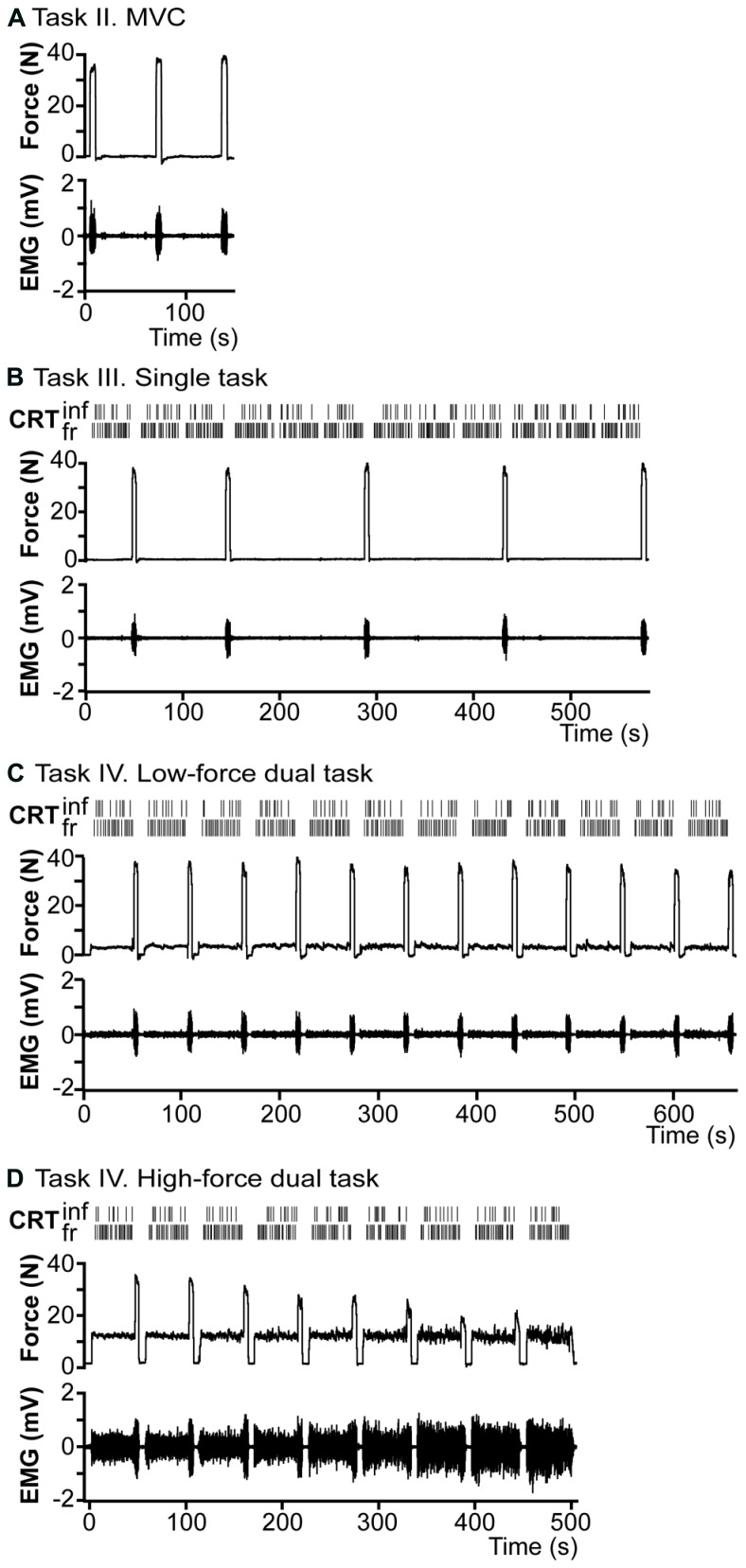
**Overview of the raw data of a representative subject. (A)** Task II, right index finger abduction force and EMG recordings of the first dorsal interosseus muscle (FDI) during maximal voluntary contractions (MVC). **(B)** Task III, single CRT-task. Subjects had to respond with either their index or middle finger to two stimuli with a different probability of presentation (frequent and an infrequent tone). Two top rows show the reactions to 12 blocks of CRT, with 5 short (5 s) MVCs between blocks of CRT. Force and EMG recordings are presented in row three and four. **(C)** Task IV, 10%-dual-task. Top two rows, show 12 blocks of CRT concurrent with right index finger abduction (force and EMG at row three and four) at 10% of cMVC, with a period of 5 s MVC after every block. **(D)** Task IV. 30%-dual-task. Similar to **(C)**, but force at 30% of cMVC, the task is maintained until task failure.

Participants came for three sessions, separated by 1 week. The first session was a practice session. All tasks were practiced in order to familiarize participants with the task and to minimize learning effects in the cognitive task. Some of the tasks were performed with fewer blocks in the practice session compared to the second and third session. In the second and third session, a dual-task at either 10 or 30% of maximal voluntary contraction (MVC) was performed. For an overview of the tasks, see **Figures 1** and **2**. Each session consisted of four tasks:

#### Task I

***Practice of CRT.*** Responding to 150 stimuli (Task Ia), followed by three blocks of 50 stimuli, with 5 s between the blocks (Task Ib). The purpose of the practice tasks was to minimize the within-session learning effect.

#### Task II

***Determination of MVC.*** Participants were asked to maximally abduct their right index finger three times for 5 s, with 60 s of rest between attempts. The maximal force of the three attempts was taken as the control MVC (cMVC), the mean rectified EMG (100 ms) around the peak force was used as the control EMG (cEMG).

#### Task III

***Single-task.*** Twelve blocks of the CRT consisting of 33 stimuli (~40 s blocks). After the first, third, sixth, ninth, and twelfth block, subjects were instructed to perform a 5 s MVC with their right FDI to acquaint subjects with the timing of the MVCs during the dual-task.

#### Task IV

***Dual task.*** Task consists of a combination of the force and CRT task. Participants were instructed to maintain a stable force level at either 10 or 30% of their cMVC in that session. All subjects were instructed to prioritize the motor task over the cognitive task in order to standardize the dual task performance. Four seconds into the force-task, the cognitive stimuli started and subjects had to respond to the stimuli with their left hand while still abducting their right index finger. Similar to the CRT (single-task), blocks of 33 stimuli were presented. After the CRT task ended, participants were asked to perform an MVC during 5 s, followed by 5 s rest. This task sequence continued for 12 blocks for the 10%-dual-task, and until task failure for the 30%-dual-task (we anticipated that most subjects would not be able to continue the 30%-dual-task for more than 10 blocks). Task failure was defined as a subject being unable to maintain the force at 30% for more than 5 s, or when the MVC did not exceed 30%.

### OUTCOME MEASURES

The main outcome measures during task II were the MVC and the maximal rectified and smoothed (100 ms) EMG. During the dual-task (task IV), mean submaximal force (% of cMVC), variability (SD) of the submaximal force, mean rectified EMG activity (% of cEMG) and MVCs (% of cMVC) were determined. Cognitive outcome measures consisted of the percentage of incorrect responses and the reaction times. The first two responses of each block were discarded because participants were informed that the first tone was always the frequent tone. Responses faster than 100 ms were counted as incorrect. Only reaction times to correct responses were used in the analyses. In order to minimize the influence of outliers, 20% trimmed means were used to assess mean reaction time values per subject, i.e., the slowest and fastest 10% of reactions on each task per subject were removed from the analysis.

### STATISTICS

In order to investigate within-session training effects, repeated measures ANOVAs were performed with Task (Task Ia, Task Ib, Task III) and Probability (frequent, infrequent) as within-subjects effects and Age group as between-subjects effects. To investigate between-session learning effects, we used the cognitive data obtained during the single CRT task. For all sessions only the first six blocks were used since the practice session consisted of six blocks only. Repeated measures ANOVAs were performed with Session and Probability as within-subjects effects and Age group as between-subjects effects. If the assumption of sphericity was not met, degrees of freedom were Greenhouse-Geisser-corrected.

The MVCs between groups were tested with a univariate ANOVA with Age group and Sex as factors; Sex was added as a between-subjects variable since it is known that men are stronger than women.

For comparison of the single- versus the dual-task performance, we used cognitive data from 12 blocks of both the single- and the 10%-dual-task. To investigate the effect of fatigue we compared the data obtained during the 10- and 30%-dual-task. The data was averaged for two time windows: the first and second half of the task. Both the cognitive and the force-related data obtained during the 10%-dual-task were averaged for the same number of blocks as for the 30%-dual-task. This was not possible for one middle-aged and two young participants, who maintained the 30%-dual-task for 13 and 15 blocks. For these participants, 12 blocks of the 10%-dual-task were used. Four young participants maintained the dual-task for only three blocks. In order to obtain a reliable estimate for the reaction time and accuracy data, the second block of data was used to calculate results for both the first (=average block 1 and 2) and second (=average block 2 and 3) part of the task. Force data was examined with repeated measures ANOVA with Task (10 and 30%-dual-task) and Time (first and second half) as within-subjects factors and Age group as between-subjects factor.

Cognitive data was examined with repeated measures ANOVA with Task and Probability as within-subjects factors, and Age group as a between-subjects factor. For comparison of the 10%-dual-task with the 30%-dual-task, Time (first and second half) was added as a within-subjects factor. For comparison of the 10%- versus the 30%-dual-task, the percentage of incorrect responses and reaction times of both dual-tasks were represented as the delta of the dual-task minus the single-task. These analyses were performed separately for both outcome measures; that is, percentage of incorrect responses and reaction times.

Statistical significance was set at alpha is 0.05, effect sizes are reported as η = SQRT(SS_factor_/SS_total_). If interaction effects were present, main effects were not explicitly described. *Post hoc* analyses (within-subject: Bonferroni corrected where appropriate; between-subject analysis: univariate ANOVAs) were performed to break down significant effects where appropriate.

## RESULTS

### TRAINING EFFECTS

#### Between sessions subjects responded faster for frequent stimuli with less errors

**FIGURE 3:**
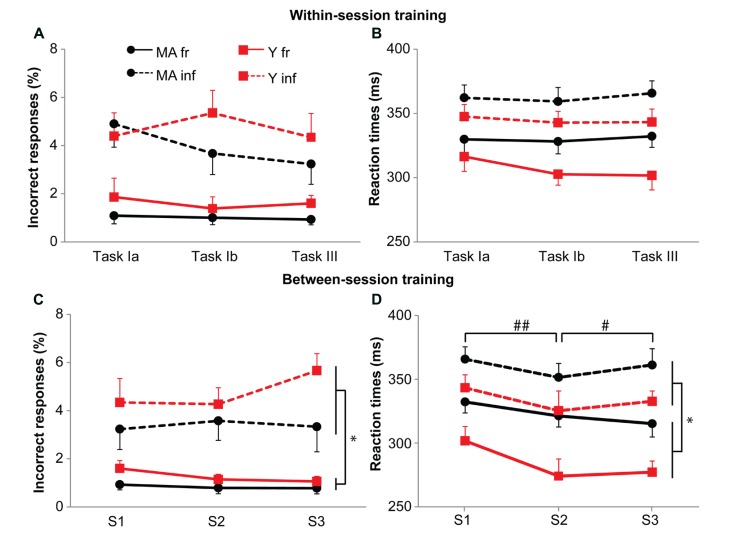
**Effect of training on accuracy (A,C) and reaction times (B,D).** Black circles represent middle-aged subjects, red squares represent young subjects. Solid lines for frequent stimuli, dashed lines for infrequent stimuli. **(A,B)** Within-session practice effects. Task Ia: 150 stimuli of the CRT task; Task Ib: 3*50 stimuli of the CRT task; Task III: 6*33 stimuli of the CRT task. **(C,D)** Between-session practice effects. Data represent first six blocks of CRT task on task III. Error bars represent SE. * shows main effects, ## shows main between session effects for frequent and infrequent stimuli, # shows between sessions effect for frequent stimuli only. Please note for the young subjects, the learning curve on reaction times for the frequent stimuli.

We used the data obtained during the cognitive single-task in the three sessions to ascertain possible differences in training effects between the two age groups (see **Figure 3**). Analysis revealed no training effects within a session for accuracy (*F*_1.7,66.2_ = 0.92, *p* = 0.39) or reaction times (*F*_2.0,77.1_ = 0.56, *p* = 0.57). Furthermore, no training effects were observed on accuracy between sessions (*F*_1.2,73.9_ = 0.62, *p* = 0.47). For reaction times, an interaction effect of Session by Probability was present (*F*_1.6,60.7_ = 10.41, *p* < 0.001, η = 0.09; **Figure 3**). *Post hoc* analyses showed that for frequent stimuli, reactions became faster from Session 1 (315 ms) to Session 2 (299 ms; *F*_1,39_ = 7.68, *p* = 0.01, η = 0.37), and from Session 2 to Session 3 (287 ms; *F*_1,39_ = 22.0, *p* < 0.001, η = 0.24). For infrequent stimuli, reaction times decreased only from Session 1 (351 ms) to Session 2 (340 ms; *F*_1,39_ = 4.7, *p* = 0.04, η = 0.30; Session 3: 335 ms). The difference in reaction times and number of errors between frequent and infrequent stimuli were present in all sessions with faster reactions and less errors for frequent stimuli (reaction times: Session 1: *F* = 111.87, *p* < 0.001, η = 0.86; Session 2, *F* = 190.39, *p* < 0.001, η = 0.91; and Session 3, *F* = 323.29, *p* < 0.001, η = 0.94; accuracy: Session 1: *F* = 52.74, *p* < 0.001, η = 0.76; Session 2, *F* = 53.51, *p* < 0.001, η = 0.82; and Session 3, *F* = 70.64, *p* < 0.001, η = 0.80). Overall, subjects were responding faster and more accurate to frequent stimuli, suggesting the presence of preparation.

### SINGLE- VERSUS DUAL-TASK PERFORMANCE

#### Middle-aged subjects responded slower to frequent but more accurate to infrequent stimuli

**FIGURE 4:**
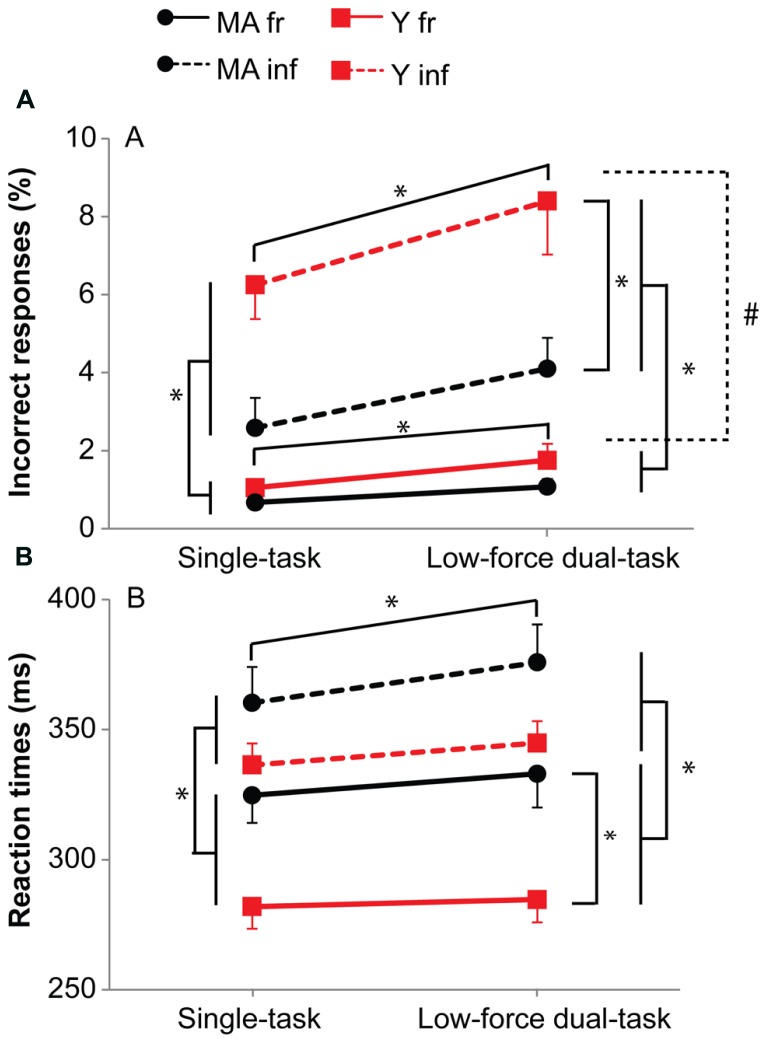
**Accuracy (A) and reaction times (B) for the single and 10%-dual-task (10% MVC).** Black circles represent middle-aged subjects, red squares represent young subjects. Solid lines for frequent stimuli, dashed lines for infrequent stimuli. Error bars represent SE. * shows main effects, # shows interaction effects. The graphs shows the difference between the two age groups with respect to preparation, i.e., faster reaction times for the frequent stimuli, but larger number of errors for the infrequent stimuli especially for the young subjects.

Besides main effects, the analysis revealed an interaction effect of Probability by Age group for accuracy (*F*_1,39_ = 7.59, *p* = 0.01, η = 0.23) and reaction times (*F*_1,39_ = 4.10, *p* = 0.05, η = 0.14). *Post hoc* analysis showed for the *infrequent stimuli* a higher number of errors for young subjects (middle-aged: 3.8%, young: 7.3%; *F*_1,39_ = 6.76, *p* = 0.013, η = 0.38), but not for the frequent stimuli (middle-aged: 1.9%, young: 3.6%; *F*_1,39_ = 1.27, *p* = 0.27; **Figure 4**). In contrast, the reaction times for *frequent* stimuli were higher for middle-aged subjects (middle-aged: 325 ms, young: 283 ms; *F*_1,39_ = 9.68, *p* = 0.003, η = 0.45), but not for the infrequent stimuli (middle-aged: 366 ms, young: 341 ms; *F*_1,39_ = 3.32, *p* = 0.076; **Figure 4**). Both middle-aged and young participants made more errors (*F*_1,39_ = 57.78, *p* < 0.001; *t*_15_ = -4.8, *p* < 0.001, *t*_24_ = -7.1, *p* < 0.001, respectively) and responded slower (*F*_1,39_ = 153.59, *p* < 0.001; *t*_15_ = -7.6, *p* < 0.001, *t*_24_ = -10.8, *p* < 0.001, respectively) on the infrequent stimuli compared to the frequent stimuli (**Figure 4**). Overall, middle-aged subjects made less errors (*F*_1,39_ = 5.81, *p* = 0.02, η = 0.36) but responded slower (*F*_1,39_ = 6.55, *p* = 0.01, η = 0.38; **Figure 4**).

#### Dual task performance resulted in more errors and slower responses for infrequent stimuli

The data showed an interaction effect of Task by Probability for accuracy (*F*_1,39_ = 4.43, *p* = 0.04, η = 0.10) and reaction times (*F*_1,39_ = 7.63, *p* = 0.01, η = 0.05). Although an increase in errors was present from the single- to the dual-task for both the frequent (single-task: 0.9%, dual-task: 1.4%; *F*_1,39_ = 4.92, *p* = 0.03, η = 0.33) and infrequent stimuli (single-task: 4.6%, dual-task: 6.5%; *F*_1,39_ = 6.22, *p* = 0.02, η = 0.37), the increase was larger for the infrequent stimuli. Reaction times were also longer for the *infrequent* stimuli in the dual- versus the single-task (single-task: 348 ms, dual-task: 359 ms; *F*_1,39_ = 15.41, *p* < 0.001, η = 0.53), but not for the frequent stimuli (single-task: 302 ms, dual-task: 307 ms; *F*_1,39_ = 2.43, *p* = 0.13).

In both the single- and the dual-task, participants made more errors (*t*_40_ = -6.9, *p* < 0.001; *t*_40_ = -7.0, *p* < 0.001, respectively) and were slower (*t*_40_ = -12.2, *p* < 0.001, *t*_40_ = -12.4, *p* < 0.001, respectively) on the infrequent stimuli compared to the frequent stimuli (**Figure 4**). Overall, the single versus dual task data demonstrated that in both tasks, young subjects responded faster to the frequent stimuli but made more errors on the infrequent stimuli than middle-aged participants did, suggesting that young subjects prepared themselves better for the frequent stimuli. The more demanding dual-task resulted in slower responses and more errors for the infrequent stimuli, without changes in reaction times for the frequent stimuli.

### 30%- VERSUS 10%-DUAL-TASK: EFFECTS OF FATIGUE ON MOTOR PERFORMANCE

#### 30%-dual-task resulted in more fatigue, but not in differences between age groups

The maximal index finger force at the start of the session was not different in the 10- and 30%-dual task session (10% session: 39.0 N, SD 10.4; 30% session: 39.4 N, SD 11.1; *p* = 0.66). Furthermore, no difference was found in MVCs between young (39.4 N, SD 10.8) and middle-aged participants (44.6 N, SD 11.5; *F*_1,37_ = 1.91, *p* = 0.18). Men were, however, stronger than women (*F*_1,37_ = 22.19, *p* < 0.001), but no interaction effect of Age group by Sex was present (*F*_1,37_ = 0.13, *p* = 0.72). The number of blocks until fatigue (task failure) did not differ between young and middle-aged participants (7.0, SD 3.4 and 7.7, SD 2.1, respectively; *F*_1,39_ = 0.52, *p* = 0.48).

**FIGURE 5:**
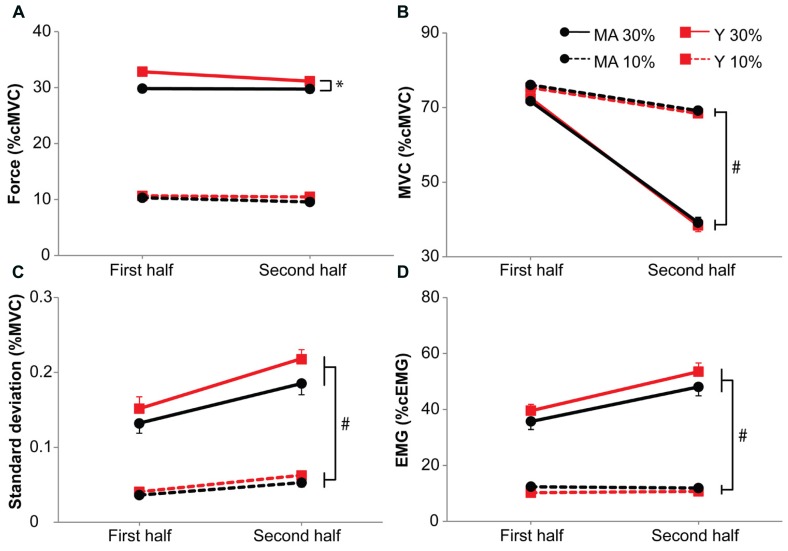
**Submaximal force (A), maximal force (B), standard deviation of the submaximal force (C) and EMG during the submaximal force task (D) during the 10 and 30%-dual-task.** Means are calculated for the first and second half of the task. Black circles represent middle-aged subjects, red squares represent young subjects. Solid lines for 30%-dual-task, dashed lines for 10%-dual-task. Error bars represent SE. * Shows main effects, # shows interaction effects. Signs of fatigue in the second half of the dual task are clearly visible for the 30%-dual-task (i.e., decline in MVC, increase in the standard deviation of the submaximal force associated with an increase in EMG).

Middle-aged participants produced marginally less force during the 30%-dual-task (mean force: 30% of cMVC) than young participants did (32% of cMVC; *p* = 0.01, **Figure 5A**). No differences were found on the 10%-dual-task (mean force: 11% of cMVC versus 10% of cMVC, for young and middle-aged participants, respectively; *p* = 0.22).

During the 30%-dual-task the intermittent MVCs declined more (33%) than during the 10%-dual-task (7%, *F*_1,39_ = 194.09, *p* < 0.001, η = 0.42), but no main effect of Age group was present (*F*_1,39_ = 0.42, *p* = 0.84, **Figure 5B**).

The standard deviation of force during the dual-task showed a stronger increase with time during the 30%-dual task (0.02% of MVC) than during the 10%-dual-task (0.01% of MVC; *F*_1,39_ = 25.15, *p* < 0.001, η = 0.16). No main effect of Age group was present (*F*_1,39_ = 0.07, *p* = 0.79, **Figure 5C**).

The EMG increased more with time in the 30%-dual-task (with 13.1%) compared to the 10%-dual-task (0.0%, *F*_1,39_ = 73.53, *p* < 0.001, η = 0.18). No main effect of Age group was present (*F*_1,39_ = 0.51, *p* = 0.48, **Figure 5D**).

In summary, data from the maximal forces, standard deviation of force and the EMG indicated that participants became more fatigued during the 30%-dual-task, but no difference was evident between young and middle-aged participants.

### 30%-VERSUS 10%-DUAL-TASK: EFFECTS OF FATIGUE ON COGNITIVE PERFORMANCE

To standardize for possible changes in performance between sessions we calculated the difference between the performance on the single- and the dual-task within the same session. Thus, the presented data are shown as deltas (dual-task data minus single-task data).

#### 30%-dual-task resulted in slower responses and a trend toward more errors with time

In the comparison between the 10 and 30%-dual-task, we found a trend toward an interaction effect of Task by Time for accuracy (*F*_1,39_ = 3.80, *p* = 0.06, η = 0.08) and a significant interaction for reaction times (*F*_1,39_ = 8.24, *p* = 0.01, η = 0.15). With time, an increase was seen in number of errors and reaction times for the 30%-dual-task (*t*_40_ = -2.6, *p* = 0.013; *t*_40_ = -4.1, *p* < 0.001, respectively), but not in the 10%-dual-task (*t*_40_ = -0.4, *p* = 0.69, *t*_40_ = -0.6, *p* = 0.56).

#### 30%-dual-task resulted in slower responses and more errors than the 10%-dual-task

**FIGURE 6:**
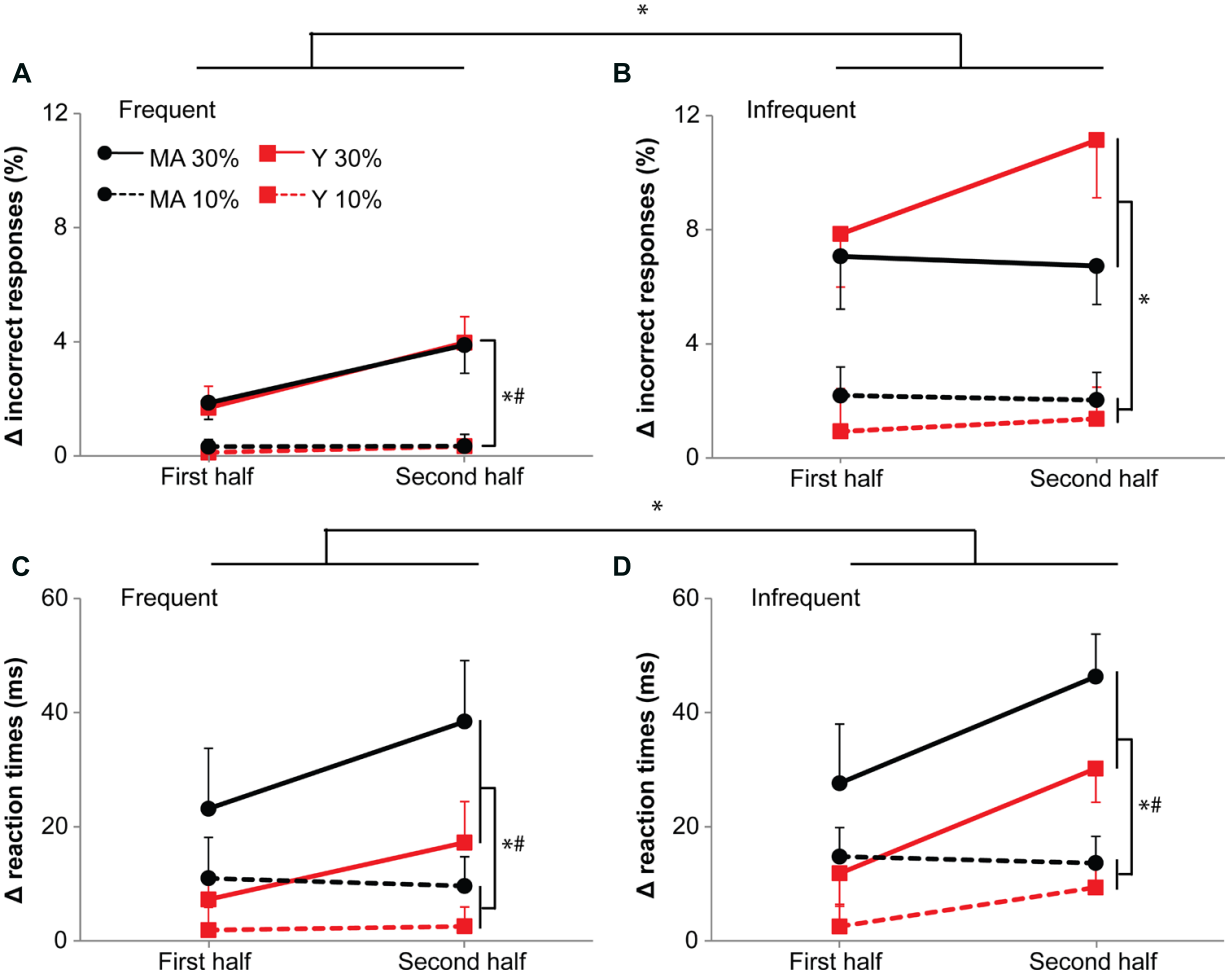
**Accuracy (A,B) and reaction times **(C,D)** during the 10 and 30%-dual-task. (A,C)** Show responses to frequent stimuli, **(B,D)** responses to infrequent stimuli. Data is represented as the difference between dual-task and single-task (delta). Black circles represent middle-aged subjects, red squares represent young subjects. Solid lines for 30%-dual-task (30% cMVC), dashed lines for 10%-dual-task (10% cMVC). Data are shown for the first and second half of the task. Error bars represent SE. * shows main effects, # shows interaction effects. Please note the increase in errors (see, however; middle-aged subjects on infrequent stimuli) and reaction times with time for the 30%-dual-task.

Slower responses (+17 and +33 ms, in the first and second half of the dual task) and more errors (4.6 and 6.4%, respectively) were made in the 30%- compared to the 10%-dual-task (+8 and +9 ms, 0.9 and 1.0%, respectively; reaction times: *F*_1,39_ = 14.88, *p* < 0.001, η = 0.35; accuracy: *F*_1,39_ = 53.41, *p* < 0.001, η = 0.43; **Figure 6**).

For accuracy, we found an additional interaction effect of Task by Probability (*F*_1,39_ = 12.52, *p* < 0.001, η = 0.19). An increase in errors between the 10- and 30%-dual-task was present for both frequent (10%-dual-task: 0.3%, 30%-dual-task: 2.8%; *F*_1,39_ = 30.24, *p* < 0.001, η = 0.49) and infrequent stimuli (10%-dual-task: 1.6%, 30%-dual-task: 8.2%; *F*_1,39_ = 35.89, *p* < 0.001, η = 0.55), the increase being larger for the infrequent stimuli.

#### Larger dual-task costs for reaction times in middle aged subjects

A main effect of Age group for reaction times (*F*_1,39_ = 5.10, *p* = 0.03, η = 0.34; **Figure 6**) shows that dual-task demands were greater for the middle-aged than the young participants.

### 30%- VERSUS 10%-DUAL-TASK: EFFECTS OF SEX ON FATIGUE EFFECTS

There are indications that sex has an effect on motor (Hicks et al., 2001; Hunter et al., 2004) and dual-task (Yoon et al., 2009) performance. Sex differences on cognitive performance have not been studied extensively, although in a large sample size (*n* = 7130), Der and Deary (2006) found an effect of sex on reaction times. Our data seemed to indicate some effects of sex; therefore, we decided to analyze the data with regard to sex differences. We examined the influence of sex by performing a mixed design repeated measures ANOVA where Sex was added as a between-subjects factor and Age (grand mean) was added as a covariate.

On the single- and dual-tasks, no difference on accuracy was found for Sex (single-task: 3.0 and 2.9%; *F*_1,38_ = 0.10, *p* = 0.75; 10%-dual-task: 3.6 and 3.5%; *F*_1,38_ = 0.10, *p* = 0.76; 30%-dual-task: 3.6 and 2.9%; *F*_1,38_ = 0.41, *p* = 0.52, for men and women, respectively), nor did we find interaction effects with Sex. We did see, however, that women responded slower than men did (single-task: 333 and 310 ms, *F*_1,38_ = 4.35, *p* = 0.04, η = 0.29; 10%-dual-task: 336 and 315 ms, *F*_1,38_ = 4.02, *p* = 0.05). For the 30%-dual-task an interaction effect of Task by Sex (*F*_1,38_ = 7.16, *p* = 0.01, η = 0.23) was found. Therefore, we decided to run the analyses on the effects of fatigue again, but separately for men and women.

#### 30%-dual-task increased the number of errors with time, especially in young men

**FIGURE 7:**
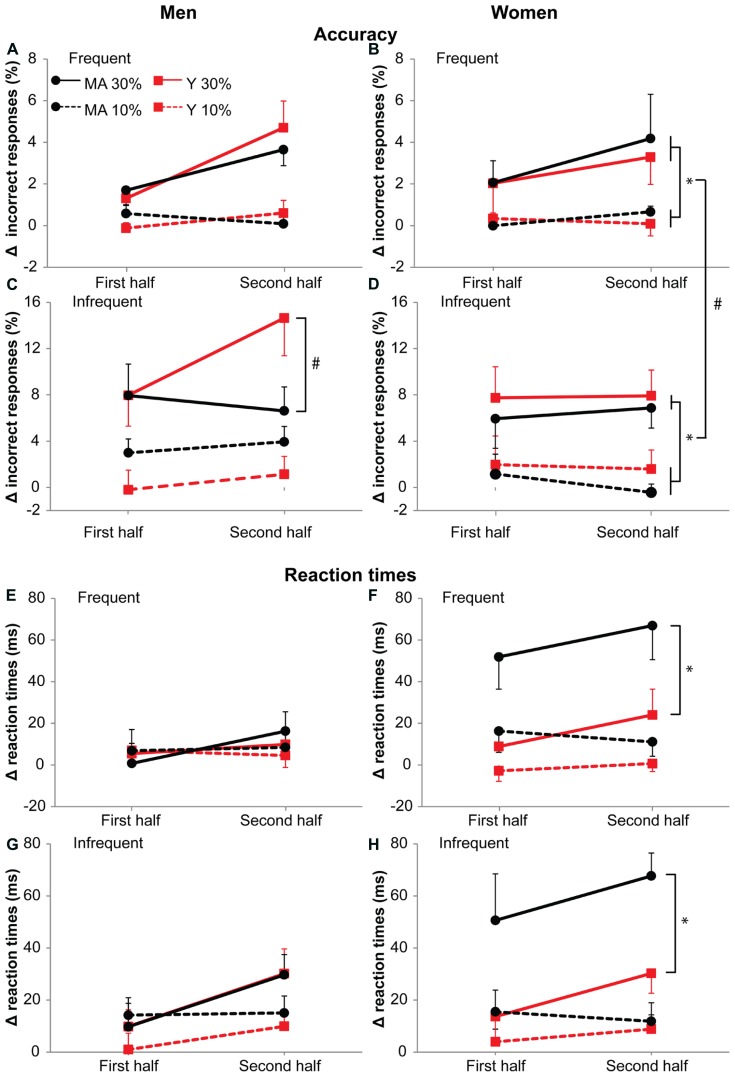
**Accuracy (A–D) and reaction times **(E–H)** during the 10 and 30%-dual-task.** Left column: men. Right column: women. **(A,B,E,F)** Show responses to frequent stimuli, **(C,D,G,H)** responses to infrequent stimuli. Data is represented as the difference between dual-task and single task (delta). Black circles represent middle-aged subjects, red squares represent young subjects. Solid lines for 30%-dual-task (30% cMVC), dashed lines for 10%-dual-task (10% cMVC). Data are shown for the first and second half of the task. Error bars represent SE. * shows main effects, # shows interaction effects. Young men showed on infrequent stimuli **(C)** an increase in errors with time on the 30%-dual-task. Middle-aged women **(F,H)** showed a large increase in reaction times from the single- to the dual-task (graphs shows deltas) during the 30%-dual-task.

For accuracy in men, we found an interaction effect of Task by Probability by Time by Age group (*F*_1,19_ = 4.96, *p* = 0.04, η = 0.08). The number of errors for the infrequent stimulus in the 30%-dual-task increased with time more for young men, as can be seen in **Figure 7**. For reaction times in men, an interaction effect of Task by Time was present (*F*_1,19_ = 5.74, *p* = 0.03, η = 0.16). At the start, the reaction times for the 10- and 30%-dual-task were similar (*F*_1,20_ = 0.001, *p* = 0.98) but the reaction times increased more during the 30%-dual-task (from +6 to +21 ms, *p* = 0.008,) than during the 10%-dual-task (+7 to +9 ms, *p* = 0.49; 10%-dual-task versus 30%-dual-task: *F*_1,20_ = 4.40, *p* = 0.05). During the dual-task, reaction times were slower for the infrequent (+15 ms) than the frequent (+7 ms) stimuli (*F*_1,19_ = 4.97, *p* = 0.04, η = 0.019).

#### 30%-dual-task increased reaction times especially in middle aged women

For accuracy in women, there was an interaction effect of Task by Probability (*F*_1,18_ = 6.22, *p* = 0.02, η = 0.17). For both stimuli, the number of errors were larger in the 30%-dual-task (frequent stimuli: 0.3 and 2.9%, *F*_1,19_ = 9.58, *p* = 0.006, η = 0.58; infrequent stimuli:1.1 and 7.1%; *F*_1,19_ = 22.44, *p* < 0.001, η = 0.74; **Figure 7**). The difference in errors between the stimuli were, however, larger in the 30%-dual-task (*t*_19_ = -4.1, *p* = 0.001) than the 10%-dual-task (*t*_19_ = -0.9, *p* = 0.366). For reaction times in women, an interaction effect of Task by Age group was present (*F*_1,18_ = 5.48, *p* = 0.03, η = 0.25). Middle-aged women showed slower responses than young women in the 30%-dual-task (+59 and +19 ms, for middle-aged and young women, respectively; *F*_1,18_ = 8.49, *p* = 0.009), but not in the 10%-dual-task (+14 and +3 ms, for middle-aged and young women, respectively; *F*_1,18_ = 3.84, *p* = 0.07). However, both Age groups showed an effect of dual-task force on reaction times (young: *t*_12_ = -2.2, *p* = 0.05, middle-aged women: *t*_6_ = -4.71, *p* = 0.003). Thus, the increase in reaction times from a 10 to 30%-dual-task was present in both young (17 ms) and middle-aged women (46 ms), but stronger in middle-aged women.

Overall, the analysis that was done separately for male and female subjects pointed to a differential effect of age on male and female adults. In middle-aged female participants, the 30%-dual-task resulted in an increase in reaction times, already at the start of the task. In men, an additional effect of age was observed on fatigue-related changes in accuracy. Men showed an increase in errors with fatigue, this increase being most pronounced in young men.

## DISCUSSION

The main findings of the present study demonstrate that already at middle-age participants: (1) responded slower but more accurate than young subjects did; (2) were less prepared for frequent stimuli than young subjects were; (3) showed an additional increase in reaction times during the 30%-dual-task. Furthermore, both young and middle-aged subjects showed a decline in cognitive performance under dual-task compared to single-task conditions and during the fatiguing dual-task, cognitive performance showed an additional decline. This decline during fatigue was not different for middle-aged compared to young participants. Pilot analyses, however, in which the data were split for sex, showed differential effects of age for men and women on dual-task performance under fatiguing conditions.

### SINGLE-TASK PERFORMANCE: EFFECTS OF AGE ON RESPONSE PREPARATION

With age, cognitive tasks become increasingly more difficult to perform (Salthouse, 1996; Der and Deary, 2006; Ren et al., 2013). Our data confirmed and extended the observation that cognitive performance, as measured by reaction times, showed a significant deterioration in middle-aged and older subjects (Smith and Brewer, 1995, 58–75 years; Yordanova et al., 2004, mean 58 years; Der and Deary, 2006, 18–81 years; Albinet et al., 2012, 61–84 years; van de Laar et al., 2012, mean 75 years).

We designed the cognitive task such that one stimulus had a higher probability (70%) to be presented than the other stimulus (30%). Consequently, after implicit learning subjects started to prepare for the frequent stimulus (Goodin et al., 1990; Miller, 1998), thereby reducing computational time and thus reducing the reaction times for the frequent stimuli. Conversely, the reaction times for infrequent stimuli increases. Furthermore, we expected that due to the preparation for the more frequent stimulus the low-probability stimulus would be more difficult and more sensitive to changes in attentional demands.

Indeed, during the practice sessions the reaction times, especially on the frequent stimuli, decreased with time, and the difference between reaction times on frequent and infrequent stimuli increased between the sessions. However, despite the training effects middle-aged subjects continued to be slower, but more accurate, than young subjects did. The age difference in reaction times was more evident in the frequent stimuli (larger difference between young and middle-aged participants for the frequent than the infrequent stimuli). On the other hand, the differences in accuracy were more evident in the infrequent stimuli (young adults making more errors than middle-aged adults did). The observed difference in reaction times between frequent and infrequent stimuli (young: 55 ms; middle-aged: 38 ms) were comparable to prior experiments (Miller, 1998: 50–88 ms in young male subjects; Eickhoff et al., 2011: 50 ms, both studies used a 20–80% probability). These results demonstrate that even after training young subjects were better prepared for the frequent stimuli. This observation may be explained by the hypothesis that young subjects rely more on proactive cognitive control and older subjects more on reactive cognitive control (Jimura and Braver, 2010).

Our data confirmed literature showing a better preparation by young subjects compared to older subjects, albeit using a different approach (Sterr and Dean, 2008; Vallesi et al., 2009; Wild-Wall and Falkenstein, 2010), and extended their results to middle-aged subjects. Preparation for a stimulus is advantageous from a behavioral point of view. The subject focuses on one response and prepares this response so that the reaction will be fast. The chance, however, that a response on the low-probability stimulus is erroneous increases. Since elderly subjects prefer accuracy above speed (Rabbitt, 1979, 62–73 years; Welford, 1988; Smith and Brewer, 1995, 58–75 years), this seemed not to be an optimum strategy for this age group. In a recent study Forstmann et al. (2011) suggested that the focus on accuracy in older subjects is not necessarily a conscious strategy but that contrary to young adults, older adults are not even capable to choose speed over accuracy. They based their hypothesis on a decline in the integrity of the cortico-striatal connections in older subjects – resulting in a lower excitability of the primary motor cortex – that showed a moderate association with speed-accuracy trade off values (Forstmann et al., 2011). Preparation also leads to additional cortical activity in motor and cognition related areas, most prominently in the prefrontal cortex (Vallesi et al., 2009; Eickhoff et al., 2011). The observation that with age these areas show already more activity to perform a motor task (Mattay et al., 2002; Ward and Frackowiak, 2003; Heuninckx et al., 2005) suggests that preparation may be less optimal in an older (and middle-aged) subject group.

### SINGLE- VERSUS DUAL-TASK PERFORMANCE

Tasks become less automatic with increasing age (Heuninckx et al., 2005; Wu and Hallett, 2005) and especially complex motor tasks require increased cortical control with increasing age (Heuninckx et al., 2005). A classical method to study the attentional control over a task is the dual-task paradigm (Pashler, 1994). We expected that, since older subjects show more cognitive involvement during the performance of a motor task (Mattay et al., 2002; Ward and Frackowiak, 2003; Heuninckx et al., 2005), adding an extra cognitive task would result in stronger detrimental dual-task effects in the middle-aged subjects, especially in response to the more complex, infrequent stimuli. Our data showed that in comparison with single-task performance, performance of the cognitive task indeed decreased during the dual-task paradigm (conform, Lorist et al., 2002). Furthermore, the effect of the dual-task was, as expected, most visible in the infrequent stimuli. The hypothesized effect of age on dual-task performance was, however, only seen in the more demanding 30%-dual-task. These increased dual-task costs with higher force levels confirmed our earlier findings (Zijdewind et al., 2006).

Previous studies found mixed results during dual-task performance. Some studies found dual-task performance to decrease with increasing age (Crossley and Hiscock, 1992; Hauer et al., 2002; Albinet et al., 2006; Voelcker-Rehage and Alberts, 2007; Fraser et al., 2010), whereas others did not (Fraser et al., 2007; Hartley et al., 2011). A meta-analysis by Verhaeghen et al. (2003) showed that age does affect dual-task performance. Nevertheless, most likely, the presence of an age effect depends on the complexity of the tasks (Crossley and Hiscock, 1992; Hauer et al., 2002; Fraser et al., 2010) and on the age of the subjects (Crossley and Hiscock, 1992). Additionally, the difference in strategy utilized by our young and middle-aged participants to perform the cognitive task is probably important. We hypothesized that the attentional reserve of the young subjects during the performance of the cognitive task would be larger and that cognitive performance in a dual-task paradigm would therefore be better maintained in young subjects. Our cognitive data, however, demonstrated that young subjects prepared more for the frequent stimuli and we propose that stronger preparation increases the load of the cognitive task, resulting in a *smaller* attentional reserve. Thus, the observation that middle-aged subjects prepared less for the frequent stimuli may result in a smaller or no difference in attentional reserve capacity between the two age groups. Still, during the dual-task paradigm, additional resources need to be utilized and since task performance deteriorated in both age groups, it is expected that the attentional requirements exceeded the attentional resources for both age groups; albeit for different reasons.

### FATIGUING DUAL TASK PERFORMANCE

The force measures did not show an effect of age. Previous experiments also demonstrated that force of a hand muscle is relatively stable and only starts to decline at high age (Doherty, 2003). With respect to fatigue and age, the literature is less consistent, but for hand muscles, most data showed no change in fatigability from young to middle-aged individuals (Chatterjee and Chowdhuri, 1991; Smolander et al., 1998; Christie et al., 2011). This is consistent with our findings, where motor performance under fatiguing conditions did not differ between the two age groups.

The data clearly showed an increase in muscle fatigue with time during the 30%-dual-task. This was illustrated by the force decline, EMG increase and increased force variability especially during the 30%-dual-task. By inducing muscle fatigue, attention needed to perform the motor task increases, as is shown in previous studies (Liu et al., 2003; van Duinen et al., 2007; Post et al., 2009) by the stronger activation of sensorimotor areas and the activation of additional (attention related) areas including the premotor and parietal areas during a fatiguing task. It was shown that by inducing muscle fatigue in young adults, the performance on a concurrent cognitive task decreased significantly (Lorist et al., 2002). Given that older individuals already allocate more attention to the motor task (Ward and Frackowiak, 2003; Heuninckx et al., 2005), we expected an additional effect of age on performance under fatiguing conditions. We did find decreased performance with increasing fatigue on a dual-task, consistent with the results of Lorist et al. (2002), but we did not find an interaction between Age, Task, and Time.

Interestingly, sex was found to affect our results. The increase in reaction times by the middle-aged women on the 30%-dual-task was the most distinct result in women. This slowness can be a result of the increase in force levels (Zijdewind et al., 2006). However, we did not expect the effect of force levels to affect women differentially. In the past, it has been shown that women were more affected by a concurrent cognitive task during the performance of a motor task (Yoon et al., 2009); while performing a secondary cognitive task, performance on a motor task decreased more in women than in men. Furthermore, in a large sample size, Der and Deary (2006) showed that women react more slowly than men do on a CRT. This is in line with our results, where women are slower already on a single-task and their reaction times on the 30%-dual-task increased more compared to men. It should be stated however, that our data was collected from only seven middle-aged women.

Men showed an effect of age group on accuracy performance with increasing fatigue, but only in the 30%-dual-task on infrequent stimuli (see **Figure 7C**). In order to maintain fast reaction times under fatiguing conditions, young men appear to prepare more, leading to more errors on the infrequent stimuli.

In summary, our results showed that age already affects cognitive performance in midlife. Especially the different strategies used by middle-aged and young adults in the reaction time task are of interest. Most of the changes are early markers of age-related changes in cognitive performance, as described by several authors for elderly subjects (Salthouse, 2000; Hartley, 2001; Der and Deary, 2006; Sterr and Dean, 2008). With increasing age, subjects tend to prioritize differently when faced with the choice of a fast or accurate response, and this prioritization remained present under dual-task conditions and even increases for young men under fatiguing conditions. Besides from a fundamental interest in the effects of age on the central nervous system, our results are also of importance from a behavioral point of view.

## Conflict of Interest Statement

The authors declare that the research was conducted in the absence of any commercial or financial relationships that could be construed as a potential conflict of interest.
